# Contribution of Autophagy-Lysosomal Pathway in the Exosomal Secretion of Alpha-Synuclein and Its Impact in the Progression of Parkinson’s Disease

**DOI:** 10.3389/fnmol.2022.805087

**Published:** 2022-02-17

**Authors:** Denisse Sepúlveda, Marisol Cisternas-Olmedo, Javiera Arcos, Melissa Nassif, René L. Vidal

**Affiliations:** ^1^Center for Integrative Biology, Facultad de Ciencias, Universidad Mayor, Santiago, Chile; ^2^Biomedical Neuroscience Institute, University of Chile, Santiago, Chile; ^3^Geroscience Center for Brain Health and Metabolism, Santiago, Chile; ^4^Escuela de Biotecnología, Facultad de Ciencias, Universidad Mayor, Santiago, Chile

**Keywords:** autophagy-lysosomal pathway, α-syn exosomal secretion, Parkinson’s disease progression, biomarker, degradation

## Abstract

Parkinson’s disease (PD) is caused by the degeneration of dopaminergic neurons due to an accumulation of intraneuronal abnormal alpha-synuclein (α-syn) protein aggregates. It has been reported that the levels of exosomal α-syn of neuronal origin in plasma correlate significantly with motor dysfunction, highlighting the exosomes containing α-syn as a potential biomarker of PD. In addition, it has been found that the selective autophagy-lysosomal pathway (ALP) contributes to the secretion of misfolded proteins involved in neurodegenerative diseases. In this review, we describe the evidence that supports the relationship between the ALP and α-syn exosomal secretion on the PD progression and its implications in the diagnosis and progression of this pathology.

## Introduction

Parkinson’s disease (PD) is the second more common neurodegenerative disease globally, and it is associated with age (Dorsey et al., 2018). The PD incidence is estimated from 5 to more than 35 new cases per 100,000 individuals, depending on demographic differences (Poewe et al., 2017). The pathology prevalence ranges from 41/100,000 individuals in the fourth decade of life to more than 1,900/100,000 among those 80 and older (Pringsheim et al., 2014). Have been described that PD is more prevalent in men (1,729/100,000, >65 years) than in women (1,644/100,000) (Pringsheim et al., 2014; Riedel et al., 2016). Although the risk of developing PD is higher in men, women have a higher mortality rate and faster clinical progression (Cerri et al., 2019).

Parkinson’s disease is a complex neurodegenerative disorder clinically characterized by bradykinesia, tremor, rigidity, later postural reflexes instability, and progressive paralysis (Jankovic, 2008). Some non-motor symptoms such as dementia, depression, anxiety, and sleep disorder may precede motor symptoms for more than a decade, affecting several neurotransmitter pathways (Langston, 2006). PD coexists with dementia in over 25% of the cases and depression in over 30% of the cases in some countries (Riedel et al., 2016). As a motor disorder, PD affects patients’ quality of life, making social interaction more difficult and worsening their financial condition due to the high medical expenses associated with the pathology.

Part of the PD symptomatology is caused by the degeneration of dopaminergic (DA) neurons of Substantia Nigra pars compacta (SNpc) and loss of the dopaminergic fibers that innervate the striatum (Dauer and Przedborski, 2003). The appearance of the first symptoms correlates with a 30% loss of dopaminergic neurons (Fearnley and Lees, 1991), indicating degeneration of the neurotransmission integrity in the basal ganglia circuits. Currently, PD has no cure, and the potential treatment to prevent or revert this pathology arises as a substantial challenge (Troncoso-Escudero et al., 2020). Therefore, it is essential to obtain a better understanding of the correlation between associated cellular mechanisms and the clinical features of PD to develop strategies to optimize the prevention, diagnosis, and treatment.

About 10% of PD cases are associated with genetic mutations (Selvaraj and Piramanayagam, 2019), which includes: (i) mutation in the *SCNA* gene that encodes for the alpha-synuclein protein (α-syn) (Bras et al., 2020), (ii) mutations in the *LRRK2* gene, which encodes leucine-rich repeat kinase 2, are a cause of autosomal dominant forms of PD (Zimprich et al., 2004; Tolosa et al., 2020), (iii) heterozygous mutations of the *GBA1*, encoding for lysosomal enzyme glucocerebrosidase (GCase), are a strong risk factor for PD and can lead to α-syn accumulation (Avenali et al., 2020). While 90% of PD cases are classified as idiopathic, the evidence indicates that the histopathology characteristic of PD is the accumulation of intraneuronal abnormal protein aggregates, including the α-syn protein. These aggregates of the amyloid type are called Lewy bodies and are constituted mainly by oligomers of α-syn and ubiquitin (Spillantini et al., 1998; Schulz-Schaeffer, 2010). Idiopathic cases show an increase of endogenous wild-type α-syn (Golbe et al., 1990; Michell et al., 2005; Shprecher et al., 2018), forming aggregates of this protein that have a toxic effect on dopaminergic neurons, triggering neurodegenerative processes (Petrucelli et al., 2002; Volpicelli-Daley et al., 2016).

Mitochondrial toxins have been identified in epidemiological studies as contributors to “sporadic” PD in humans. In this context, animal and *in vitro* models used to study PD are based on the administration of neurotoxins, generating oxidative stress and mitochondrial dysfunction. 6-hydroxydopamine (6-OHDA), 1-methyl-4-phenyl-1,2,3,6-tetrahydropyridine (MPTP), paraquat (PQ; 1, 10-dimethyl-4,40-bipyridinium), and rotenone are conventionally used in PD modeling (Chia et al., 2020), as they can be uptaken by DA neurons through dopamine transporters, inhibiting complex I of the mitochondrial electron transport chain, leading to ATP depletion, increasing reactive oxygen species, and ultimately resulting in neuronal death (Betarbet et al., 2002; Devi et al., 2008; Potashkin et al., 2010). PQ, a commonly used herbicide, shares structural similarities with MPP+, the active metabolite of MPTP. PQ crosses the blood-brain barrier, generates reactive oxygen and nitrogen species (ROS/RNS), and causes the loss of SNpc DA neurons in animal models (Castello et al., 2007). In rats, chronic rotenone exposure leads to α-syn aggregation, DA neurodegeneration, and behavioral defects (Hoglinger et al., 2003). This toxin induced the cytosolic accumulation of α-syn through the *de novo* synthesis, rather than a reduction of degradation by chaperone-mediated autophagy (CMA), suggesting a mechanism independent from lysosomal degradation (Sala et al., 2013). Indeed, rotenone regulates α-syn phosphorylation, reducing protein phosphatase 2A (PP2A) activity (Wang et al., 2016).

Classical pharmacological therapies for PD patients are dopamine precursors as levodopa, L-dopa, and L-3,4-dihydroxyphenylalanine. Other treatments include dopamine agonists such as amantadine, apomorphine, pramipexole, and monoamine oxidase inhibitors (MAO) or catechol-*O*-methyltransferase (COMT). The sustained administration of these drugs induces a “wearing-off phenomenon” and additional psychomotor, cardio-cerebrovascular, and hormones regulation problems (Cacabelos, 2017). Novel biotherapies, as natural products, should achieve dopaminergic protection to avoid neurodegeneration, enhancing dopaminergic neurotransmission. However, the cellular and molecular events involved in PD must be broadly explored to design and develop efficient treatments (Solayman et al., 2017; Wang et al., 2017; Troncoso-Escudero et al., 2020).

## Alpha-Synuclein (α-Syn): A Hallmark in Parkinson’s Disease

α-syn protein is expressed at high levels in the central nervous system (CNS), specifically neurons. It is found in presynaptic terminals as a monomeric, unfolded, and soluble protein (Maroteaux et al., 1988), bound with high affinity to the membranes of synaptic vesicles (Burre et al., 2010). α-syn was described in neuromuscular junctions (Askanas et al., 2000), suggesting other cellular functions in addition to its activity in the CNS. Although α-syn is enriched in synaptic boutons, which sprout from axons of different neurochemical phenotypes, α-syn is not present in all synaptic terminals. In agreement, not all terminals accumulate the protein in neurodegenerative disorders (Totterdell et al., 2004). Furthermore, the expression of α-syn is not limited to the nervous system. This protein is present in the cerebrospinal fluid (CSF), in plasma (El-Agnaf et al., 2003; Forland et al., 2018), as well as is expressed in the erythropoietic lineage cells (Nakai et al., 2007) and peripheral lymphocytes (Kim et al., 2004).

To clarify the α-syn function, knockout mice for the *SCNA* gene were generated. Although knockout mice were viable and fertile, with a lack of spontaneous neurodegeneration signs, this model displays alterations in activity-dependent dopamine release from axons in the striatum (Abeliovich et al., 2000). In addition, the triple knockout mice lacking the three variants of syn (α, β, and γ) were generated, showing no neurodegeneration (Greten-Harrison et al., 2010). However, it was possible to observe synapse-structure modifications and a decrease in the synaptic terminal size in an age-dependent manner (Greten-Harrison et al., 2010). Lack of α-syn in the transgenic mice model showed less mobilization of glutamatergic vesicles (Gureviciene et al., 2007) and increased the expression levels of proteins involved in vesicle traffic, such as SNAREs, synapsins, and complexines (Greten-Harrison et al., 2010). In addition, it has been described that the participation of α-syn in vesicle homeostasis is Ca^2+^-dependent (Lautenschlager et al., 2018). Overall, these data suggest a direct physiologic role of α-syn in synaptic transmission in the CNS, especially in the dopamine system.

Different factors can trigger α-syn aggregation, including point mutations (Narhi et al., 1999; Rutherford et al., 2014), truncations (Li et al., 2005), posttranslational modifications of α-syn (Duda et al., 2000; Fujiwara et al., 2002; Lazaro et al., 2014), and wild-type SCNA gene duplication or triplication (Singleton et al., 2003; Fuchs et al., 2007). An increase in somatic copy number of the *SNCA* gene in CNS neurons, especially from the SN region, was reported in a cohort of PD patients, contributing to the sporadic α-syn accumulation (Mokretar et al., 2018). However, it is still unknown what prompts the accumulation of wild-type α-syn into toxic aggregates. Evidence indicates that toxic α-syn conformers can act as seeds for the misfolding and aggregation of the native protein. For instance, α-syn preformed fibrils (PFFs), synthetically produced, were added in neuronal cultures and taken up inside cells, recruiting α-syn endogenous into protein aggregates (Luk et al., 2012a,b). The inoculation of PFFs into the brain of young adult A53T *SNCA* mice (overexpressing mutated human α-syn) generated *in vivo* aggregates and PD-like symptoms in mice (Luk et al., 2012b). Moreover, species of α-syn isolated from A53T transgenic mice induce aggregation of α-syn in primary neuronal cultures (Colla et al., 2018), indicating a potential trigger role of α-syn aggregates on wild-type soluble α-syn.

Nevertheless, not only protein interactions determine the status of α-syn aggregation. α-syn contains a lipid-binding domain that allows its binding to vesicles at the presynaptic terminal (Lashuel et al., 2013). However, in pathological conditions or modified lipids composition, this interaction can potentiate conformational changes in α-syn protein, prompting it to aggregation (Marschallinger et al., 2020). Recently, it was reported that the lipid alteration in membrane compartments, as instability of lipid raft microdomains, promoted by aging, and neurotoxins, as the MPTP, could affect α-syn aggregation (Galvagnion, 2017; Canerina-Amaro et al., 2019). Caveolins, a subgroup of lipid rafts, act as scaffolding proteins that recruit other proteins and lipids, leading to colocalization and interaction of proteins involved in vesicular transport, signal transduction, and receptor trafficking (Hanzal-Bayer and Hancock, 2007). The central protein controlling caveolae formation is caveolin-1 (Cav-1). Cav-1 is widely expressed in the central and peripheral nervous systems (Boulware et al., 2007), regulating neurotrophin signaling pathways and synaptic remodeling (Bilderback et al., 1999; Suzuki et al., 2004). In addition, Cav-1 modulates neurotransmitter receptor signaling (Bhatnagar et al., 2004; Francesconi et al., 2009).

aveolin-1 is also involved in the aging process. Since Cav-1 expression is upregulated in old rat brain and aged human cortex (Park et al., 2000; Kang et al., 2006), suggesting that overexpression of Cav-1 may induce aging phenotypes (Wheaton et al., 2001; Lee et al., 2015). Some evidence suggests that scaffold proteins such as Cav-1 may be involved in the pathogenesis of several neurodegenerative disorders, including PD (Hashimoto et al., 2003; Benarroch, 2007). Age-related expression of Cav-1 may affect the cell-to-cell transmission of α-syn, contributing to the pathogenesis of PD (Ha et al., 2021). Cav-1 overexpression facilitated the uptake of α-syn into neurons and the formation of additional Lewy body-like inclusion bodies (Ha et al., 2021). Immunoprecipitation experiments demonstrated that the double mutant alpha-synuclein protein (A30P/A53T) interacts with Cav-1 present in both cytoplasmic and inner membrane extracts of the mouse brain, suggesting that the double mutation of α-syn increases the affinity for Cav-1 in the cytosol. These results suggest a direct interaction between Cav-1 and α-syn under non-physiological conditions (α-syn overexpressed or α-syn mutated) (Madeira et al., 2011). Furthermore, colocalization experiments using SH-SY5Y cells demonstrate that α-syn and caveolin interact directly and mediate endocytosis and colocalize to a lesser extent along the endocytosis pathway with early endosome antigen 1 (EEA1) and Rab7-positive late endosomes (Fakhree et al., 2021). EEA1 is an early endosomal Rab5 effector protein that has been implicated in the docking of incoming endocytic vesicles before fusion with early endosomes (Fakhree et al., 2021), and Rab7, a member of the Rab family of small GTPases, is a ubiquitously expressed protein that plays a vital role in the regulation of the trafficking, maturation, and fusion of endocytic and autophagic vesicles (McCray et al., 2010).

A recent work using human iPSC-derived cerebral organoids found that 3D-cultures from donors carrying homozygous *APOE4* allele presented aggregation of α-syn, loss of synaptic integrity, and impairment on lipids metabolism, resulting in accumulation of lipid droplets (Zhao et al., 2021). APOE4 isoform is a known risk factor for late-onset Alzheimer’s disease (AD) development. In this study, researchers also reported a boosted interaction of APOE4 itself with α-syn in postmortem brain samples from Lewy bodies disease patients (Zhao et al., 2021), confirming a link between lipid metabolism and α-syn aggregation. As previously mentioned, heterozygosis variants in the gene encoding GCase (*GBA1*) represent a significant PD genetic risk factor (Avenali et al., 2020). Indeed, about 10% of PD patients present mutations in the gene that codifies to GCase (Sidransky et al., 2009). GCase is a lysosomal enzyme that catalyzes the hydrolysis of the glycolipid glucosylceramide. Homozygous mutations in *GBA1* cause Gaucher’s disease, the most prevalent recessively inherited lysosomal lipid storage disease, characterized by neurodegeneration and peripheral symptoms (Han et al., 2020). *GBA1* variants associated with PD present a decreased enzymatic activity, resulting in the accumulation of the substrate glucosylceramide, as shown in CSF samples from PD patients (Huh et al., 2021). Accumulated glucosylceramides were reported to promote wild-type α-syn aggregation in *in vitro* studies (Taguchi et al., 2017). Of note, a small-molecule modulator (activator) of GCase reduces pathological α-syn aggregates and restores lysosomal function in PD patient midbrain neurons (Mazzulli et al., 2016b). In a bilateral correlation, α-syn aggregation also causes impairment on GCase activity and lysosomal dysfunction (see below).

The overexpression of α-syn selectively induced apoptotic programmed cell death in primary dopamine neurons (Zhou et al., 2000), neuroblastoma cell lines, and hippocampal primary neurons (Mahul-Mellier et al., 2015). The causal relationship between α-syn aggregation and cellular toxicity was investigated by assessing the effect of inhibiting fibrillization on α-syn-induced cell death. It was reported that exogenous α-syn fibrils bind to the plasma membrane and act as nucleation sites for the formation of endogenous α-syn fibrils, promoting the accumulation and internalization of the aggregates that finally turn on the activation of both the extrinsic and intrinsic apoptotic cell death pathways in cellular models (Mahul-Mellier et al., 2015).

It has been described that secreted α-syn can be internalized by neighboring cells *via* endocytosis (Desplats et al., 2009), demonstrating the cell-to-cell transmission of α-syn accumulation and providing evidence of the pathological mechanism to explain PD progression and other synucleinopathies (Desplats et al., 2009; Hansen et al., 2011). Recent works also showed α-syn transference cell-to-cell through the formation of tunneling nanotubes (TNTs; Abounit et al., 2016; Dieriks et al., 2017). The α-syn uptake by cells depends on the fibrillization (Luk et al., 2009) and oligomeric (Lee et al., 2008) state of α-syn. Oligomers of α-syn have more significant cytotoxicity in recipient cells than soluble monomers of α-syn (Desplats et al., 2009; Emmanouilidou et al., 2010). It was reported that a single intrastriatal injection of synthetic α-syn fibrils initiates a pathological α-syn transmission sufficient to cause PD-like neurodegeneration in non-transgenic mice (Luk et al., 2012b). Furthermore, extracellular α-syn has been shown to activate microglia and astroglia, enhancing neurodegeneration, indicating a cell non-autonomous mechanism (Zhang et al., 2005; Klegeris et al., 2006).

An abundance of synaptic vesicle-related proteins like CD9 (exosomes), Clathrin, AP-2 complex, and dynamin (clathrin-mediated endocytosis), dynein, dynactin, and spectrin (retrograde transport), synaptosomal-associated protein 25, vesicle-associated membrane protein 2, and syntaxin-1 (synaptic vesicle fusion) are present in α-syn-containing protein inclusions purified from *post mortem* brain tissues from dementia with Lewy bodies (DLB) patients (McCormack et al., 2019). Different models of intercellular transmission of α-syn, not mutually exclusive, have been proposed, such as the α-syn cellular release, movement, and uptake, by different mechanisms, including exocytosis, exosomes, TNTs, glymphatic flow, and endocytosis (Valdinocci et al., 2017). In this regard, different types of vesicles are released from the cells depending on the metabolic and homeostatic cellular status into the extracellular space (extracellular vesicles, EVs), such as exosomes. EVs act as a shuttle for cargo delivery between cells, participate in cell-to-cell communication, and have a potential pathogenic role in the cell-to-cell transmission of toxic aggregated proteins in a neurodegenerative disease context.

## Exosomal α-Syn Secretion in Parkinson’s Disease and Its Impact on Disease Progression

Exosomes are small vesicles (40–100 nm in diameter) released into the extracellular space by various cell types, including neurons, astrocytes, microglia, and lymphocytes. Exosomes are generated from multivesicular bodies (MVB) that, after fusion with the plasma membrane, releases intraluminal vesicles – exosomes – containing membrane components, proteins, lipids, and microRNAs (Kowal et al., 2014; Hessvik and Llorente, 2018). Moreover, this EV population can be detected in body fluids such as blood, urine, and CSF (Thery et al., 2006; Keller et al., 2011).

Increasing evidence suggests that the secretion of α-syn, and oligomeric species, is associated with membrane vesicles, as exosomes (Alvarez-Erviti et al., 2011; Danzer et al., 2012; Emmanouilidou and Vekrellis, 2016). The secretion of exosomal α-syn is a calcium-dependent mechanism (Emmanouilidou et al., 2010). The mechanism of exosomes internalization is not entirely decoded, and it seems to depend on the type of recipient cells (Fruhbeis et al., 2013; Nanbo et al., 2013; Svensson et al., 2013; Tian et al., 2014). The pathways caveolin-dependent, clathrin-dependent, and macropinocytosis are not involved in the internalization of exosome-associated oligomeric α-syn (Delenclos et al., 2017). Furthermore, heparin sulfate proteoglycans (HSPGs), transmembrane, and lipid-anchored cell surface receptors modulate the internalization exosomes containing Aβ monomer (Kanekiyo et al., 2011) and α-syn recombinant fibrils (Holmes et al., 2013). However, in contradictory results, another group reported that the deficiency of HSPG did not attenuate the up-taking of α-syn exosomes (Delenclos et al., 2017), suggesting that this pathway is not critical for the α-syn oligomers internalization.

Neurons secrete α-syn by non-canonical cellular pathways that may involve the participation of chaperones UPS19 and DNAJ/HSC70 complex (Lee et al., 2016; Bieri et al., 2018). Although the mechanism of exosomal α-syn secretion and uptake has not been elucidated, it is more apparent that the secreted vesicular α-syn is readily internalized compared to free α-syn oligomers (Delenclos et al., 2017; Gustafsson et al., 2018), conferring toxicity on the neighboring cells (Emmanouilidou et al., 2010; Danzer et al., 2012).

Exosomes may provide a catalytic environment for nucleation of α-syn aggregation (Grey et al., 2015). Vesicles containing-α-syn have been shown to increase the oligomerization status of α-syn (Lee et al., 2005; Grey et al., 2015), and oligomers negatively impact cellular health more than monomers. α-syn species with presumably lost physiological functions or altered aggregation properties may shift the cellular processing toward vesicular secretion. Fluorescent protein tags on the N-terminus of α-syn alter intracellular dynamics (Goncalves and Outeiro, 2013) and induce vesicular secretion (Jang et al., 2010). N-terminal protein tags on α-syn lead to altered membrane-binding properties and may form particularly pathogenic and stable forms of aggregated α-syn that could increase cell-to-cell spreading (Gustafsson et al., 2018).

**FIGURE 1:**
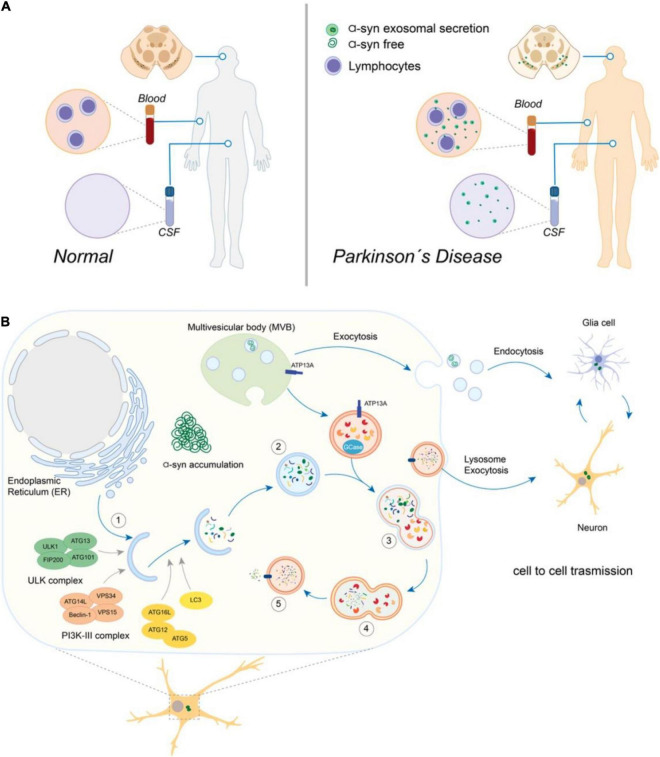
Crosstalk between the autophagy-lysosomal pathway (ALP) and the exosomes secretion in Parkinson’s disease. **(A)** The presence of soluble free or exosomes-containing α-synuclein (α-syn) derived from Substantia Nigra can be detected in cerebrospinal fluid (CSF) and blood samples from Parkinson’s disease patients, potentially contributing to the early disease’s diagnosis and progression monitoring. **(B)** Overview of the autophagy-lysosomal pathway (ALP) and exosomes secretion. In (1), the formation of the initial membrane that will originate the double-vesicle autophagosome (2) depends on several complex proteins’ actions (shown in different colors). (3) The fusion of autophagosomes with lysosomes is a final step of the pathway, originating the autolysosome (4), where the substrates are finally degraded into their monomeric components that can be recycled back to the cytosol (5). Multivesicular bodies (MVB) originate the exosomes vesicles, which are secreted by exocytosis and participate in the cell-to-cell transmission of α-syn (neurons and glia cells).

A minor fraction (0.1–2%) of secreted α-syn are associated with EVs, whereas most of the protein can be found free in the extracellular space (Danzer et al., 2012; Shi et al., 2014). Even though the EV-associated fraction of extracellular α-syn is slight, such vesicles are considered biologically active (van Niel et al., 2006) and molecules in this environment could be more efficiently delivered to other cells (Subra et al., 2010). Interestingly, the exosomal α-syn levels of neuronal origin in plasma correlate significantly with motor dysfunction, a parameter of the severity of the disease (Shi et al., 2014), highlighting the exosomes containing α-syn as a potential biomarker of PD (Figure 1A). α-syn and DJ-1, also known as Parkinson’s disease protein 7 (PARK7), an antioxidant, transcriptional co-activator, and molecular chaperone, presence in plasma neural-derived exosomes were significantly higher in PD patients (Zhao et al., 2018). Recently, it was described that exosomes derivated from saliva also contain α-syn and may be used as a potential biomarker in PD (Cao et al., 2019).

Is the secretion of exosomal α-syn an intercellular transmission mechanism that increases toxicity in the brain?, or could it be a cellular protective response against the intracellular accumulation of α-syn? This response has not been elucidated, and there is controversial literature about it. In this context, it has been described that exosomes isolated from brain tissue of patients with DLB injected into the brain of wild-type mice generate the misfolding of the endogenous α-syn protein (Ngolab et al., 2017). Notable, CSF exosomes from PD patients induce oligomerization of α-syn in a reporter cell line in a dose-dependent manner (Stuendl et al., 2016). About the secretion and uptake of α-syn *via* EVs in cultured cells, it has been reported that disease-causing mutants, as A53T α-syn, displayed increased association with EVs (Gustafsson et al., 2018). It has been described that γ-syn, another protein family member of synucleins, can be oxidized and initiate α-syn aggregation. γ-syn secreted in exosomes from neuronal cells can be transmitted to glial cells and cause the aggregation of intracellular proteins (Surgucheva et al., 2012).

Interestingly, pramipexole, an agonist of the dopamine receptor family, is used as a treatment for PD patients. After 12 weeks of pharmacological therapy, the patient’s motor performance was statistically improved, and the α-syn content in serum exosomes was lower after the treatment (Luo et al., 2016). Although these results propose a correlation between the α-syn content in serum exosomes and motor symptoms, the mechanism to explain it is still unknown.

Not only neurons would participate in the transmission of α-syn exosomal since microglia also can capture exosomes from the plasma of patients with PD. α-syn induces an increase of exosomal secretion by microglia, and these exosomes showed a high level of MHC class II molecules and TNF-α (Chang et al., 2013). More recently, it was described that the secretion of exosomal human α-syn from the microglia could facilitate its aggregation (Guo et al., 2020) and propagation, possibly through dysregulation of autophagy (Xia et al., 2019).

On the other side, on physiological conditions, it is possible to detect monomers of α-syn, which are degraded by the ubiquitin-proteasome system (UPS; Bennett et al., 1999) and the chaperone-mediated autophagy (CMA; Cuervo et al., 2004; Vogiatzi et al., 2008; Mak et al., 2010). Oligomers, however, are efficiently degraded by the autophagy-lysosomal pathway (ALP; Ebrahimi-Fakhari et al., 2011). Both processes, exosomes secretion of α-syn and degradation by ALP, occur in a coordinate balance (Fussi et al., 2018; Figure 1B). Nevertheless, the mechanism involved in the balance of autophagy and α-syn exosomal secretion in neurons has not been elucidated.

## Impact of Autophagy-Lysosomal Pathway in Parkinson’s Disease Progression

Autophagy (derived from the Greek words for “self” and “eating”) is an evolutionarily conserved lysosomal pathway that digests long-lived proteins, protein aggregates, stress RNA granules, and abnormal cytoplasmic organelles. Based on the type of substrate, mode of cargo recognition, transport, and delivery to the lysosome, three types of autophagy have been described: microautophagy, CMA, and macroautophagy in PD (Martinez-Vicente and Cuervo, 2007; Kenney and Benarroch, 2015). During microautophagy, the cargo is taken into the lysosome or late endosome through its membrane invagination, being quickly degraded in the lysosomal lumen (Frake et al., 2015; Bento et al., 2016). In the CMA, the cargo containing an aminoacidic sequence binds to cytosolic chaperones, is recognized and imported into the lysosomal lumen by a receptor on the lysosomal membrane (Kaushik and Cuervo, 2008). Macroautophagy (hereafter referred to as autophagy or autophagy-lysosomal pathway) is a highly regulated mechanism that forms a double-membrane vesicle called the autophagosome to isolate the cargo that will be degraded (Bento et al., 2016). After maturation, autophagosomes fuse with lysosomes to degrade their content by the activity of lysosomal acid hydrolases (Figure 1B). Lysosomes’ proper function is central to concluding several convergent pathways, including autophagy and endocytosis. Under basal conditions, autophagy is an active quality control process that prevents metabolic and oxidative stress in the cell by degrading aggregated proteins and damaged or dysfunctional organelles. Starvation-induced autophagy is a cellular response to nutrient deprivation that recycles macromolecules to offer substrates for metabolism (Mizushima et al., 2008).

In a pathological context, it has been found that selective autophagy contributes to the clearance of misfolded proteins involved in neurodegenerative diseases such as tau, SOD1, and α-syn (Vidal et al., 2014; Frake et al., 2015). Moreover, evidence has demonstrated a link between PD and mitophagy (selective mitochondrial autophagy). Mitophagy is mediated by binding selective autophagy receptors simultaneously to ubiquitinated proteins in the mitochondria surface and proteins from the autophagy machinery, such as the LC3-II family proteins (Pickles et al., 2018; Conway et al., 2020). The most studied mitophagy pathway is dependent on two proteins, PTEN-induced kinase 1 (PINK1) and Parkin (Narendra et al., 2010). In this pathway, PINK1 accumulates on depolarized mitochondria, triggering the translocation of Parkin from the cytosol, eliciting the ubiquitination of several mitochondrial proteins, including mitofusin 1 and 2 (MFN1 and MFN2), translocase of outer membrane 20 (TOM20), and voltage-dependent anion-selective channel 1 (VDAC1; Bayrhuber et al., 2008; Wang et al., 2011; Sarraf et al., 2013). Notably, mutations in *PINK1* and *Parkin* are associated with familial parkinsonism, while the loss of PINK1 function induces oxidative stress and mitophagy (Valente et al., 2004; Dagda et al., 2009). Moreover, the parkinsonian neurotoxin MPP+ (the active metabolite of MPTP) induces autophagy and mitophagy depending on autophagy proteins ATG5, ATG7, and ATG8, but independently of the protein Beclin 1 (Chu et al., 2007).

Autosomal dominant mutations in the gene *LRRK2* encoding the protein leucine-rich repeat kinase 2 are among the most common causing familial PD (Zimprich et al., 2004). Mutations in *LRRK2* have been shown to reduce mitochondria trafficking in rat neurons (Godena et al., 2014; Hsieh et al., 2016), impair the mitophagy activity in PD-derived cells (Bonello et al., 2019; Wauters et al., 2020), and increase aggregation of α-syn in mice models and human iPSC-derived dopaminergic neurons (Bieri et al., 2019). In a recent work, researchers studied α-syn spreading levels in CSF from patients carrying different DLB and PD mutations using an α-syn real-time amplification assay (Brockmann et al., 2021). Interestingly, CSF samples from patients harboring mutations in *PINK1* or *Parkin* did not show positive α-syn seeding activity. However, CFS samples from *LRRK2* PD patients (78%) showed an elevated α-syn positivity, only exceeded by CSF samples from DLB (100%) or PD (93%) patients carrying *GBA1* mutations (Brockmann et al., 2021). Notably, this higher α-syn seeding activity in CSF from *GBA1* patients was associated with lower levels of proteins related to α-syn clearance, including autophagy, lysosomal function, and endocytosis pathways in the same samples, suggesting *GBA1* mutations promote a negative correlation between α-syn accumulation and degradation pathways, mainly associated with lysosomal dysfunction (Brockmann et al., 2021). This work also found decreased levels of proteins from the UPS and neurosecretion processes in CSF from *GBA1*-carrying patients, which increase the urge to elucidate the mechanisms involved in this broad reduction in degradation pathways proteome (Brockmann et al., 2021) and to check if this data is reflected in CNS samples.

The failure of the protein quality control systems, especially lysosomal-dependent degradation, promotes the accumulation of α-syn (Desplats et al., 2009). Heterozygous mutations in the *GBA1* gene encoding lysosomal enzyme GCase are strong risk factors for PD (Avenali et al., 2020). GCase is an N-glycosylated protein synthesized and transported in vesicles from the ER-to-Golgi apparatus, where it is correctly folded through a maturation process before reaching the lysosomes. α-syn aggregation generated by wild-type *SNCA* triplication in PD patients iPSC-derived dopaminergic neurons was reported to disrupt the ER-GA trafficking by inhibiting the SNARE protein ykt6 (Cuddy et al., 2019), depleting lysosomes from acid hydrolases, and increasing the accumulation of insoluble immature GCase in ER (Stojkovska et al., 2021). Interestingly, using a pharmacological enhancer of ER proteostasis plus a farnesyltransferase inhibitor (FTI), which restores ykt6 activity, was reestablished GCase maturation and lysosomal activity, becoming a promising therapeutic strategy for future studies in synucleinopathies (Stojkovska et al., 2021).

Recent works have suggested that the secretion of exosomes containing α-syn could result as a protection mechanism against the blockage of autophagy-dependent α-syn clearance (Fussi et al., 2018). In particular, the silencing of ATG5, a key protein involved in the extension of the phagophore membrane in autophagic vesicles (Pyo et al., 2005), increases the secretion of α-syn *via* exosomes, which are associated with a decrease in cell death α-syn induced (Fussi et al., 2018). In accordance, the inhibition of lysosomal function in α-syn overexpressing neural cell lines generated an increase of exosomal secretion of α-syn, promoting a cell-to-cell transfer of α-syn (Alvarez-Erviti et al., 2011). Other evidence also shows that the ALP inhibition reduces intracellular α-syn while increasing the secretion of smaller oligomers, exacerbating the uptake, inflammation, and cellular damage (Poehler et al., 2014). Moreover, has been reported a secretion of aggregated α-syn by exosomes and Rab11a-associated pathways and by membrane shedding (Poehler et al., 2014). It was confirmed that the ALP inhibition promotes the release and transmission of α-syn *via* EVs with a hybrid autophagosome-exosome phenotype, increasing the ratio of extracellular α-syn/intracellular α-syn and its association with EV in neuronal cells (Minakaki et al., 2018). GCase loss-of-function was also associated with α-syn secretion. Studies in transgenic mice harboring the human mutation A53T in *SNCA* found that inhibition of GCase increases the exosome-associated α-syn oligomers release (Papadopoulos et al., 2018). The plasma exosomal/total α-syn ratio is associated with GCase activity, and it correlates with severity (motor deficiency) in PD patients (Cerri et al., 2018; Johnson et al., 2020), proposing the link between lysosomal dysfunction with increased exosome secretion. Similar results were reported in fibroblasts derived from PD patients with or without *GBA1*, in which defective GCase activity increased the release of exosomes (Cerri et al., 2021). Isolated exosomes from these cells caused increased levels of phospho-α-syn in SH-SY5Y recipient cells, overexpressing wild-type α-syn (Cerri et al., 2021). Interesting, this effect was not due to a seeding effect since fibroblasts are α-syn-free. The researchers hypothesize that fibroblast-derived from patients harboring *GBA1* mutations promote changes in the lipid composition of recipient cells, which may account for the increased phospho-α-syn, posttraductional modification that increases the formation of insolubleα-syn forms (Canerina-Amaro et al., 2019). Besides releasing exosomes, MVBs can be eliminated through the ALP by a direct fusion with lysosomes or autophagosomes (Fader et al., 2008; Vanlandingham and Ceresa, 2009; Szatmari et al., 2014; Teixeira et al., 2021). α-syn itself can disturb the ALP activity, promoting potential positive feedback to its secretion. Notably, α-syn fibrils have been shown to impair lysosomes’ morphology from inside the organelle lumen, reducing the ALP-dependent clearance of aggregates and defective organelles. Moreover, lysosomes filled with α-syn fibrils can be transferred to neighboring cells through TNTs or secretion vesicles, contributing to the disease’s spread (Dilsizoglu Senol et al., 2021). There is evidence for a loop between the lysosome and α-syn proteoforms (Wildburger et al., 2020).

Some genes encoding proteins involved in intracellular vesicle trafficking and lysosome transport are risk genes associated with PD (Abeliovich and Gitler, 2016; Mazzulli et al., 2016a). For example, Kufor-Rakeb syndrome (KRS) is caused by an autosomal recessive mutation in the *PARK9* gene encoding ATP13A2 (transmembrane lysosomal type 5 P-type ATPase protein) characterized by juvenile-onset parkinsonism. Interestingly, a mutation in this gene was described in a Chilean family patient for the first time (Ramirez et al., 2006). *PARK9* encodes a lysosomal ATPase involved in cation homeostasis, and its loss of function leads to lysosomal dysfunction (Gitler et al., 2009; Kong et al., 2014; Tsunemi et al., 2014). Interestingly, in *Caenorhabditis elegans* and dopamine cell culture models, it was described that ATP13A2 would have a protective role against the accumulation of misfolded α-syn and cellular toxicity (Gitler et al., 2009). However, the overexpression of ATP13A2 increases the release of exosomes, promoting the secretion of α-syn in primary cortical neurons (Tsunemi et al., 2014). The evidence suggests that the ATP13A2 protein regulates the release of α-syn *via* EVs through the modification of the biogenesis of exosomes by a functional interaction with the lysosomal sorting complex required for transport (ESCRT; Tsunemi et al., 2014). Additionally, the enhanced secretion of exosome-associated α-syn may explain the increased viability in neurons of the SNpc in sporadic PD patients by overexpressing ATP13A2 (Kong et al., 2014). A recent work has shown that PD mutations in *ATP13A2* increase α-syn intracellular accumulation by impairing lysosome exocytosis using iPSC-derived neurons from PD patients (Tsunemi et al., 2019). The mechanism by ATP13A2 modulates lysosomal exocytosis is by mediating Ca^2+^ homeostasis in these organelles. Interestingly, a pharmacological agonist of the lysosomal Ca^2+^ channel, TRPML1, recovers lysosomal exocytosis, correcting α-syn secretion defects and decreasing intracellular accumulation in ATP13A2 patient neurons (Tsunemi et al., 2019).

What is the contribution of neighboring cells of neurons in PD? Microglia isolated from adult mice, in contrast to microglia from young mice, display phagocytosis deficits of free and exosome-associated α-syn oligomers (Bliederhaeuser et al., 2016). The neuronal α-syn secreted by exosomes or lysosomal exocytosis is partially endocytosed by astrocytes, which contribute to reducing the α-syn spread between neurons. Indeed, degradation of α-syn is more efficient in astrocytes than neurons (Tsunemi et al., 2020). However, the iPSC-derived astrocytic protection against the α-syn accumulation and propagation is partially lost by *ATP13A2* mutations, resulting in the increased accumulation and propagation of α-syn between neurons (Tsunemi et al., 2020), suggesting that astrocytic lysosomal dysfunction indirectly contributes to the α-syn neuronal pathology.

Moreover, impaired biogenesis of MVBs by a dominant-negative mutant of vacuolar protein sorting 4 (VPS4) interferes with the lysosomal targeting of α-syn and facilitates α-syn secretion (Hasegawa et al., 2011). The hypersecretion of α-syn in VPS4-defective cells was restored by the functional disruption of recycling endosome regulator Rab11a. VPS4, a master regulator of MVBs sorting, may serve as a determinant of lysosomal targeting or extracellular secretion of α-syn (Hasegawa et al., 2011). Another member of the endosomal protein sorting, VPS35, is also associated with PD (Vilarino-Guell et al., 2011). VPS35 is part of the retromer complex, which mediates the endosome-to-Golgi recovery of membrane proteins. The VPS35 D620N mutation causes an autosomal-dominant form of PD, and the cells expressing the mutant form have impaired autophagy. The defects in autophagy can be explained in part by the abnormal traffic of the transmembrane autophagy protein ATG9A (Zavodszky et al., 2014).

Another traffic protein associated with PD is Secretory Carrier Membrane Protein 5 (SCAMP5), a regulator of membrane trafficking enriched in the brain, identified as an autophagy inhibitor that promotes exosomal secretion of α-syn (Yang et al., 2017). SCAMP5 is a novel coordinator of autophagy and exosome secretion, induced under protein stress by Bafilomycin A1 to clear toxic proteins *via* the exosomes rather than ALP (Yang et al., 2017).

**FIGURE 2:**
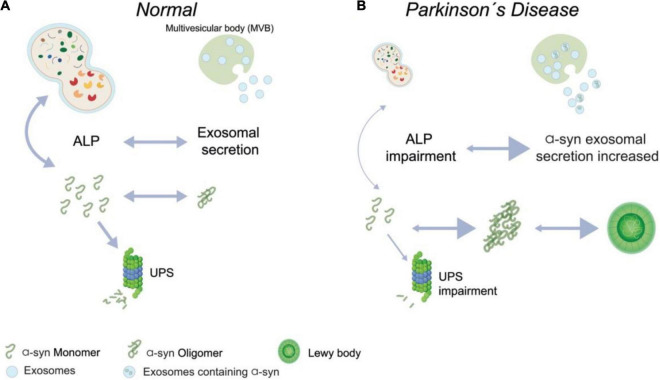
Contribution of autophagy-lysosomal pathway and α-syn secreted in Parkinson’s disease. **(A)** In normal conditions, it is possible to detect monomers of alpha-synuclein (α-syn), which are degraded by Ubiquitin Proteasome System (UPS), and oligomers of α-syn are efficiently degraded by Autophagy-Lysosomal (ALP) pathway or can be secreted by exosomes. Both degradation and secretion processes occur in a coordinate balance. **(B)** On Parkinson’s Disease condition, abundant oligomers and fibrils of α-syn are formed, and it less degraded by autophagy due to an impairment of this pathway, and it is possible to observe an increased secretion of α-syn by exosomes.

All these results support the idea that exists a connection between autophagy, lysosomal homeostasis, and α-syn exosomes secretion on the PD progression (Xu et al., 2018). Moreover, autophagy modification (gain and loss function) impacts exosomes released into the extracellular space *in vitro* (Hu et al., 2020). Although, the full mechanism underlying these processes *in vivo* and the effect on the progression of the PD remains poorly understood (Figure 2). Overall, several lines of evidence propose that a correction in lysosomal function can boost dopaminergic neurons’ survival in PD, avoiding α-syn aggregation. However, it is open to whether the increase in the α-syn secretion, by exosomes or other vesicles kinds, can result in a progression spread of the disease in an *in vivo* long-term study.

## Clinical Aspects of Autophagy-Lysosomal Pathway and Parkinson’s Disease Progression

As mentioned, oligomers and fibrils α-syn degradation is mediated by autophagy, connecting the role of lysosomes to the etiology/progression of PD (Webb et al., 2003; Lee et al., 2004). Concerning the above, the expression of genes from the autophagy pathway [UNC- 51- like kinase (ULK) 3, autophagy-related (Atg) 2A, Atg4B, Atg5, Atg16L1, and histone deacetylase 6] were evaluated in peripheral blood mononuclear cells (PBMCs) of patients with PD. Researchers observed a decrease in the expression of autophagy regulatory components in patients with PD, while they reported an increase of α-syn protein levels in PBMCs compared to controls (Miki et al., 2018). However, the comprehensive mechanisms of the dynamic interaction of ALP-MVBs for the secretion of α-syn *via* EVs have not been fully elucidated. It has been recently described that a significant percentage of the proteins detected in tissue-purified Lewy bodies from DLB patients and cytoplasmic glial inclusions (CGI) of oligodendrocytes from multiple systemic atrophy (MSA) patients are synaptic vesicle proteins, including CD9 associated with exosomes (McCormack et al., 2019). This fact suggests that the misfolding or accumulation of α-syn, characteristic of synucleinopathies, contributes to the vesicle-mediated transport of these protein inclusions (McCormack et al., 2019). A recent study demonstrated that the inhibition of dynamin-related protein 1 (Drp1) improved both mitochondrial function and autophagic flux in experimental models of α-syn (Fan et al., 2019). The following key step is to determine if the Drp1 inhibition confers neuroprotection through the abolished autophagic impairment induced by α-syn in *in vivo* models of PD.

Several pharmacological agents targeting ALP components, especially lysosomal function, are active research topics in preclinical and clinical phases to PD and other synucleinopathies. They include the previously mentioned FTI, which restores the SNARE ykt6 activity, reestablishing lysosomal hydrolases maturation, and finally, the lysosomal activity (Stojkovska et al., 2021) pharmacological agonists of lysosomal Ca^2+^ channel, TRPML1, which has been shown to restore lysosomal exocytosis, enhancing α-syn secretion and decreasing accumulation in *ATP13A2* patient iPSC-derived neurons (Tsunemi et al., 2019); or the ambroxol, a cough syrup approved by the FDA since 1971, which has been reported to reduce α-synuclein levels *in vitro* and *in vivo* (Migdalska-Richards et al., 2016), and to increase GCase expression and activity (Migdalska-Richards et al., 2017). Ambroxol was shown to restore lysosomal exocytosis (Magalhaes et al., 2018) and promote ER folding. Recently, ambroxol treatment in PD patients harboring or not *GBA1* mutations has shown promising results regarding α-syn secretion in CSF (Mullin et al., 2020). However, although these drugs are promising hopes, they need to be tested in different mutations associated with PD, considering the broad clinical and physiological variability between them (Cerri et al., 2018; Johnson et al., 2020).

## Conclusion and Perspectives

This review summarized the antecedents that demonstrate altered autophagy in PD and some evidence that proposes a link between ALP components and the exosomal secretion of α-syn. To elucidate the mechanisms that explain the relation between ALP and the secretion of EVs in PD is still a field in study. However, some clues of the crosstalk between exosomes and autophagy have been proposed [review in Xu et al. (2018), Gudbergsson and Johnsen (2019), Buratta et al. (2020), Xing et al. (2021)].

The origin of extracellular vesicles can offer additional information. For example, mitochondrial-derived vesicles are a candidate as biomarkers in body fluids of PD patients may provide clues to understand the association between mitochondrial dysfunction and systemic inflammation in PD (Picca et al., 2019). Interesting proposals are in the therapy field based on autophagic degradation and exosomal secretion. The development of an α-syn nano-scavenger for PD capable of stimulating nuclear translocation of TFEB (master regulator of autophagy), promoting autophagy and calcium-dependent exosome secretion for the clearance of α-syn (Liu et al., 2020). While α-syn expression can be reduced by antisense oligonucleotides (ASOs), the big challenge is delivering ASOs efficiently and safely into the neurons. Exosomes can be a safe and highly effective ASO delivery method (Yang et al., 2021). Promising ALP and exosomal secretion research are developed on *in vitro* models. However, further validations in animal models and physiology-related conditions are required.

Recently, *in vitro* study reported that proteins from SARS-CoV-2, which causes COVID-19, interact with α-syn, speeding up the formation of amyloid plaques (Semerdzhiev et al., 2021) open an interesting research field associating virus infections with PD or other neurodegenerative diseases development.

## Author Contributions

DS, MC-O, JA, MN, and RV wrote and edited the manuscript. DS and RV prepared the figures. DS, MN, and RV planned the manuscript. All authors contributed equally to the critical reading of the final manuscript, including figures.

## Conflict of Interest

The authors declare that the research was conducted in the absence of any commercial or financial relationships that could be construed as a potential conflict of interest.

## Publisher’s Note

All claims expressed in this article are solely those of the authors and do not necessarily represent those of their affiliated organizations, or those of the publisher, the editors and the reviewers. Any product that may be evaluated in this article, or claim that may be made by its manufacturer, is not guaranteed or endorsed by the publisher.
